# The Use of Expanded Polystyrene and Olive Stones in the Manufacture of Lightweight Bricks: Evaluation of Their Properties and Durability

**DOI:** 10.3390/ma16041330

**Published:** 2023-02-04

**Authors:** María López Gómez, Giuseppe Cultrone

**Affiliations:** Department of Mineralogy and Petrology, Faculty of Sciences, University of Granada, Avda. Fuentenueva, s/n, 18002 Granada, Spain

**Keywords:** expanded polystyrene, olive stones, bricks, sustainable bricks, physical properties, durability

## Abstract

This paper studies the effects of using 20, 40 and 60% vol. of either expanded polystyrene (EPS) or olive stones as additives in the manufacture of handmade bricks. The bricks were made using clayey earth from Viznar (Spain) and were fired at 950 °C. The effects of the additives on the mineralogical, textural and physical properties of the fired bricks were analysed, focusing mainly on possible changes in their pore system, thermal insulation, compressive strength, colour and salt crystallisation resistance. From a mineralogical point of view, the bricks made with olive stones had a lighter red colour due to their lower hematite content. As expected, the samples made with these additives had greater porosity and better thermal insulation. However, they also had lower compressive strength to the point that the only samples that met the recommended criteria for general construction work were those with 20% vol. EPS, while those with 40% vol. EPS met the criteria to be used as lightweight bricks. Both additives improved the resistance of the bricks to decay by salt crystallisation.

## 1. Introduction

There are several review papers [1,2,3] and research studies [4] dealing with the use of organic and inorganic waste products as additives in brick manufacture. Al-Fakih et al. [1] specified that waste products could partially or totally replace the clayey material in brick production as long as they reach the required standards. Murmu and Patel [2] noted that the use of agricultural and municipal wastes brings some advantages, such as a reduction in the firing temperature, which is helpful in saving energy. Zhang [3] categorised the bricks produced with wastes according to their elaboration method by firing, cementing and geopolymerisation and emphasising the importance of immobilising contaminants that may be present in the waste. Sutcu et al. [4] determined that the use of marble powder increases the sample porosity and the thermal insulation of the resulting bricks. The use of waste materials as additives for brick production has two main objectives: on the one hand, it reduces the amount of clayey earth necessary for the manufacture of bricks, and on the other, it helps to recycle the waste materials. The first factor is of the utmost importance, as clay is a non-renewable resource, and its overexploitation could lead to future shortages and negative impacts on the environment [5]. For its part, waste recycling is vital if we want to achieve the important social objective of a circular economy, in which the current huge amounts of waste produced must be reduced to a minimum. Research into the possible use of other waste products as additives in brick production has shown that these additives sometimes bring about specific changes in the mineralogy and physical properties of the bricks. For example, the addition of volcanic ash has been shown to make bricks more resistant to decay [6], while the addition of waste glass increases their mechanical resistance [7]. Furthermore, the addition of organic matter, such as rice husk and paper pulp, generally increases the porosity of the final products [8,9].

The waste products used in this paper, namely expanded polystyrene (EPS) and olive stones, vanished during the firing process, so increasing the porosity of the bricks. This improves the thermal insulation of the bricks and makes them considerably lighter. Potential benefits include reduced fossil fuel consumption for heating buildings and easier transportation of bricks. 

EPS is commonly used as packaging material and for thermal insulation in construction. At the end of its useful life, it can be mechanically recycled, fired to produce energy [10] or chemically treated to recover its constituent monomers [11]. However, we are still a long way from recycling all the EPS produced. In fact, the Association for European Manufacturers of Expanded Polystyrene (EUMEPS) has pledged to increase EPS recycling rates to 46% by 2025 [12]. To this end, research is being conducted into the possible use of EPS as an additive in the production of construction materials such as lightweight mortars [13] or bricks. Although little research has yet been conducted on the use of EPS, promising results have been obtained in various studies in which samples were produced using varying amounts of additive and different firing temperatures [14], different percentages and sizes [15] or by mixing EPS with other materials [16]. 

For their part, olive stones are one of the waste materials produced during olive oil production. Another olive oil waste, pomace, has been studied a little more extensively as an additive in the production of lightweight bricks [17]. Several other papers have been published about the use of other wastes, such as olive leaves and olive branches [18], wastewater from olive oil extraction [19], olive stones [20,21] and pomace ashes [22,23]. Spain is the world’s leading producer of olive oil, and the region with the largest cultivated area is Andalusia. Andalusian olive stones and pomace are currently being used as a source of energy through their combustion; however, due to the vast amount of waste generated (515,705 tonnes/year [24]), not all of them are being used.

The papers mentioned above focus on the properties of these bricks as thermal and acoustic insulators. However, they did not perform an exhaustive characterisation of the porous system using hydric tests, nor did they test the durability of the new bricks over time or the colour changes after firing. The aim of this study is to compare the effects of the addition of olive stone and EPS in the mineralogy, texture, porosity, thermal conductivity, colour and mechanical resistance of handmade bricks. This paper also discusses the effects of these additives on the durability of the resulting bricks.

## 2. Materials and Methods

### 2.1. Making the Brick Samples

The raw material used to make the bricks comes from clayey Pleistocene levels that outcrop 3 km from Viznar in the Granada Basin (Andalusia, Spain). They are quarried for use in a local factory that makes bricks and other ceramic products. The Granada Basin is located over the contact area between the internal and external zones of the Baetic Cordillera and started as a marine basin in the Burdigalian before becoming continental during the Latest Tortonian—Earliest Messinian. This continentalisation led to the formation of lacustrine and fluvial deposits containing, above all, conglomerates, silts and clay [25]. The dendritic material that filled this basin came from the surrounding mountains, such as Sierra Nevada and Sierra Arana, which played a special role in the formation of the north-eastern deposits [26,27]. The sediments from Sierra Arana may explain the presence of carbonates in Viznar’s clayey earth. 

The EPS was obtained from packaging material and reduced to a size of about 1 mm in diameter. The olive stones were obtained from a supplier that sells them as domestic heating fuel. They were milled in the laboratory down to fragments with a maximum diameter of 1.5 mm. The bricks were handmade using a wooden mould, measuring 16 × 12 × 4 cm, instead of an extruder. The clayey earth was kneaded with water, and the resulting paste was placed in the mould and then pressed down by hand. Once the mould was full, the surface was levelled off using a ruler. The unfired bricks were then demoulded and cut to produce smaller samples of 4 × 4 × 4 cm. Seven types of bricks were manufactured. One of them was made without additives as a control sample (labelled as R). The others were manufactured with the addition of 20%, 40% and 60% volume of either EPS (labelled as P2, P4 and P6, respectively) or olive stones (labelled as H2, H4 and H6). For comparative purposes, Table 1 shows the mass percentage corresponding to the volume of additive added and the amount of water used in the manufacture of the bricks. Note that, due to the density difference (700 kg/m^3^ for olive stones and 20 kg/m^3^ for EPS), the mass percentage of olive stones was higher than the mass percentage of EPS, even though the same volume percentage was used in both additives. After a drying period of 3 weeks, all the brick samples were fired at 950 °C in a Hobersal JM 22/16 electric oven. This temperature was chosen because it is commonly used in brick manufacture. The bricks were initially fired at 100 °C for one hour to remove any remaining moisture. After that, the temperature in the oven was increased by 3 °C/min until the selected temperature of 950 °C was reached. During firing, a burning smell was noticed in the samples that contained olive stones. After 3 h at 950 °C, the oven was switched off, although the bricks were not taken out of the oven until the next day. This allowed them to cool slowly, preventing the development of cracks due to β-α quartz transition at 573 °C [28]. The whole process took 24 h. 

**Figure 1:**
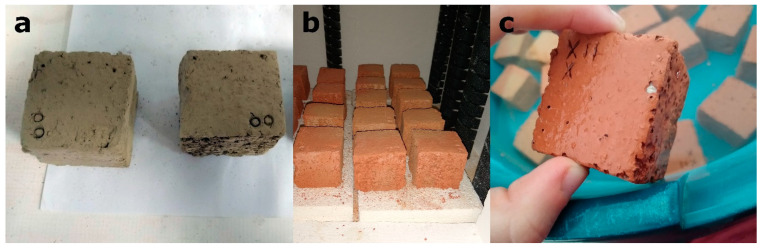
Elaboration of the brick samples: (**a**) unfired cut samples; (**b**) fired samples in the electric oven; (**c**) samples immersed in water to avoid “lime blowing” phenomenon.

During the brick manufacturing process, the higher the amount of additive used, the lower the level of water consumption (Table 1). This is an important advantage given that water is a vital and scarce natural resource. After firing, samples were immersed in water to avoid “lime blowing” [29], a phenomenon that can lead to the formation of fractures in fired bricks due to the hydration of the lime grains. This process occurs in bricks manufactured with clay that contains carbonates, such as the clay from Viznar. In macroscopic terms, the bricks with additives had more porous and irregular surfaces, and the samples with olive stones had a less reddish colour. The samples with additives appeared to be lighter than the control samples. Figure 1 illustrates the elaboration process of bricks.

### 2.2. Analytical Techniques

#### 2.2.1. Chemical Properties, Mineralogy and Texture

The chemical composition of the clayey material and the fired bricks was studied by X-ray fluorescence (XRF) using a PANalytical Zetium compact spectrometer. This technique was used to measure the weight percentage of the major oxides and the loss on ignition (LOI). The samples were milled using an agate mortar prior to the analysis.

The mineral composition of the samples was determined using X-ray diffraction (XRD) on disoriented powders, using a PANalytical X’Pert diffractometer under the following working conditions: CuKα (λ = 1.5405 Å) radiation, 45 kA voltage, 40 mA current, 4 to 70° 2θ exploration range and 0.01° 2θ/s goniometer speed. The samples were milled with an agate mortar prior to the analysis. The diffractograms were interpreted using the HighScore v.4.8. software (Malvern Panalytical, Malvern, UK) and the PDF2 database. In order to identify the clay minerals in more detail, oriented aggregate samples were prepared and treated with ethylene glycol.

To characterise the texture of the samples, a thin section of each brick was prepared and observed under a CarlZeiss Jenapol-U polarised optical microscope. In addition, carbon-coated fragments of the samples were observed and analysed at higher magnification through an FEI Quanta 400 Environmental Scanning Electron Microscope (ESEM), which was coupled with an xFlash 6/30 detector for Energy Dispersive X-ray (EDS) microanalysis.

#### 2.2.2. Study of the Porous System

The porous system of the brick samples was studied by means of free and forced water absorption [30] and drying tests [31]. These tests allowed us to calculate the drying index (D_i_); the saturation coefficient (S); the apparent (ρ_a_) and real densities (ρ_r_); the open porosity (P_o_), according to RILEM [32] and EN 772-4 [33] standards; and the degree of pore connection (A_x,_ [34]). 

Mercury intrusion porosimetry (MIP) was used to complete the information about the porous system of the bricks and determine their pore size distribution. For this test, pores within a range of 0.002–200 μm were studied using a Micromeritics Autopore V 9620 porosimeter. The test was performed on one fragment per brick sample. Prior to the test, the samples were dried in an oven at 60 °C for a week.

#### 2.2.3. Compactness, Colour and Thermal Conductivity

To evaluate the compactness of the samples, an ultrasound test was performed following the ASTM D2845-08 [35] standard. A Controls 58-E4800 UPV tester was used to measure the P-wave velocity (Vp) through cubic samples in the three orthogonal directions. 

The uniaxial compression test was carried out to determine the mechanical strength of the bricks. For this purpose, an Instron 3345 press was used. Due to the small number of samples, the test was performed on 5 samples instead of the 6 suggested in the EN 1926 [36] standard. In order to obtain plane and parallel surfaces, they were smoothed with a cutting disk.

In order to quantify the colour of the bricks and how the type and amount of additive can influence colour change, the samples were measured by means of a Minolta CM-700d spectrometer using an illuminant D65. The results were presented in the CIE L*a*b* system, where L* is the value for lightness, and a* and b* are the chromatic coordinates. In order to determine the colour differences between the samples with and without additives, the following equation was used [37]:

(1) $$\Delta E*=\sqrt{{\left(\Delta L\right)}^{2}+{\left(\Delta a*\right)}^{2}+{\left(\Delta b*\right)}^{2}}$$

The degree of thermal insulation of the bricks was determined using infrared (IR) thermography with a Flir T440 thermographic camera. The samples were placed on a hot plate at 50 °C for 30 min, and IR photographs were taken every 30 s.

#### 2.2.4. Durability by Salt Crystallisation

In order to evaluate the durability of the bricks, a salt crystallisation test was performed according to the UNE-EN 12370 [38] standard using a 14% Na_2_SO_4_ × 10H_2_O solution. Once the 15 cycles test was over, the samples were washed several times in deionised water to remove all the remaining salts from the pores and fissures and to determine how much weight the bricks had lost during the test.

## 3. Results

### 3.1. Chemical Properties, Mineralogy and Texture

The results of the XRF analysis are presented in Table 2. The concentration of major oxides is not affected by the presence of additives in the fired bricks. This is because EPS is almost totally composed of air, while the olive stone is mainly composed of C and O, being both consumed during firing. The clayey earth has a higher LOI than the fired bricks. This may be due to the dihydroxylation of phyllosilicates, the CO_2_ released from the carbonates and organic matter combustion. Logically, after firing at 950 °C, the LOI falls significantly and is slightly lower in bricks without additives (R). The fired bricks are rich in silica (53%) and have high concentrations of alumina (17%) and calcium (11%).

**Figure 2:**
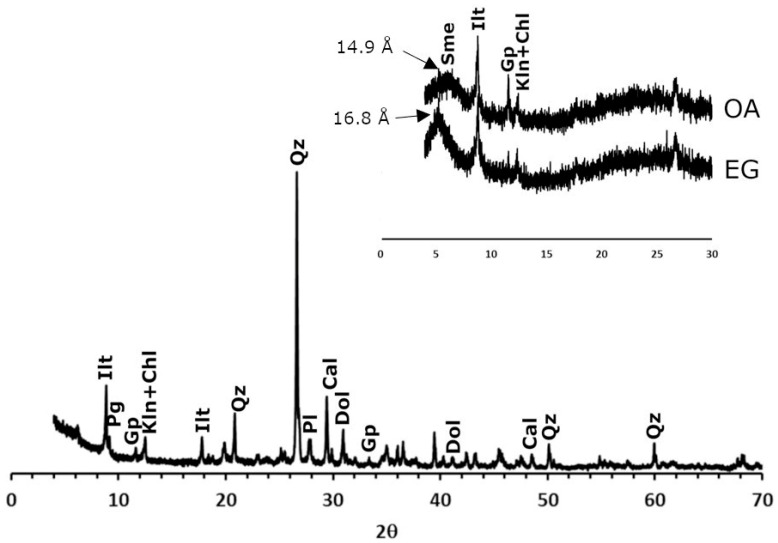
X-ray diffraction patterns of the clayey earth from Viznar. Ilt: illite; pg: paragonite; chl: chlorite; kln: kaolinite; qz: quartz; pl: plagioclase; cal: calcite; dol: dolomite; gp: gypsum; sme: smectites. Abbreviations suggested by Warr [40]. In the inset: an oriented aggregate sample of the clay fraction without treatment (OA) and after treatment with ethylene glycol (EG).

Regarding the mineralogy of the clayey earth (Figure 2), quartz, phyllosilicates (illite, paragonite, chlorite and kaolinite), plagioclase, gypsum and carbonates (calcite and dolomite) were identified. The high percentages of SiO_2_ and Al_2_O_3_ identified through XRF are mainly due to the presence of quartz and phyllosilicates that are abundant in the clayey earth. Carbonates are a characteristic component of the clay from Viznar [29], and, along with the gypsum, they are the main explanation for the calcium percentage obtained in XRF. This raw material does not normally contain gypsum, and its presence here is probably due to slight contamination with another clayey earth (from a nearby quarry in Jun) that contains this sulphate [39] and is also used in the factory that supplied the raw material. The presence of smectites was detected in a detailed analysis of the clay fraction through the aggregate-oriented samples and their treatment with ethylene glycol.

**Figure 3:**
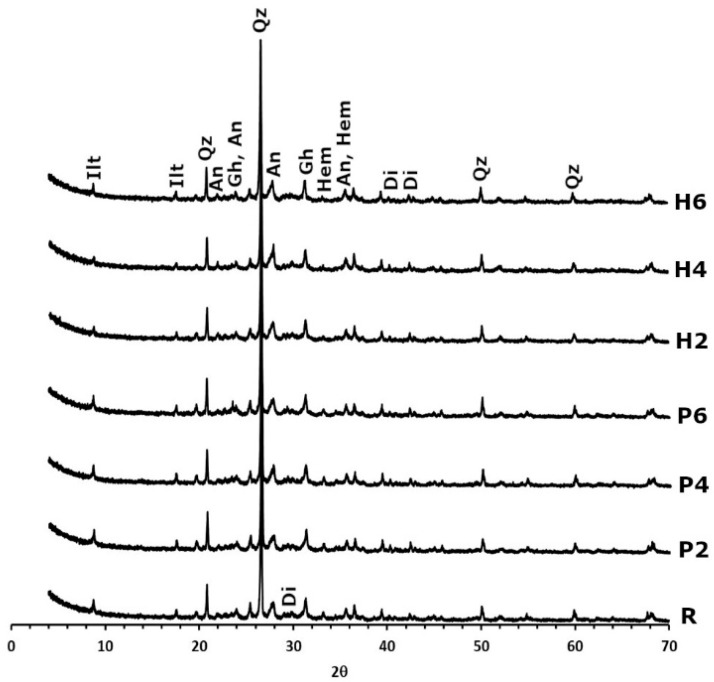
X-ray diffraction patterns of fired bricks without waste (R), with EPS (P2, P4 and P6) and with olive stones (H2, H4, and H6). Ilt: illite; qz: quartz; an: anorthite; gh: gehlenite; hem: hematite; di: diopside. Abbreviations suggested by Warr [40].

Regarding the mineralogy of the bricks (Figure 3), after firing at 950 °C, carbonates and phyllosilicates can no longer be detected. The only exception is illite, which remains in a dehydroxylated phase. Quartz can be found in the same quantities, and new phases such as gehlenite, diopside, hematite and anorthite also appear. The formation of gehlenite (Ca_2_Al_2_SiO_7_) is due to the decomposition of calcite from the raw material and its reaction with phyllosilicates to form this sorosilicate at temperatures of over 800 °C [29]. Similarly, the decomposition of dolomite into Ca and Mg ions and their reaction with quartz leads to the formation of diopside (CaMgSi_2_O_6_) [29]. The decomposition of chlorite and illite results in the crystallisation of hematite [41], while the presence of anorthite can be explained by the gradual enrichment of the calcium of the plagioclases in the clayey earth [42].

The diffractograms of the samples made with added EPS show that these bricks have the same mineralogical composition as the control sample. However, the addition of olive stones results in a decrease in hematite content. Considering that the samples have the same amount of Fe, the only possible explanation is that the firing of the organic matter, which was more abundant in the samples with olive stones, consumed a higher amount of oxygen in the oven, thus preventing part of the hematite from forming (Figure 3). This also explains why these samples are not as red as the control samples (see Section 2.1 making the brick samples), as the red colour in bricks is linked to the formation of hematite in an oxidising atmosphere [43].

**Figure 4:**
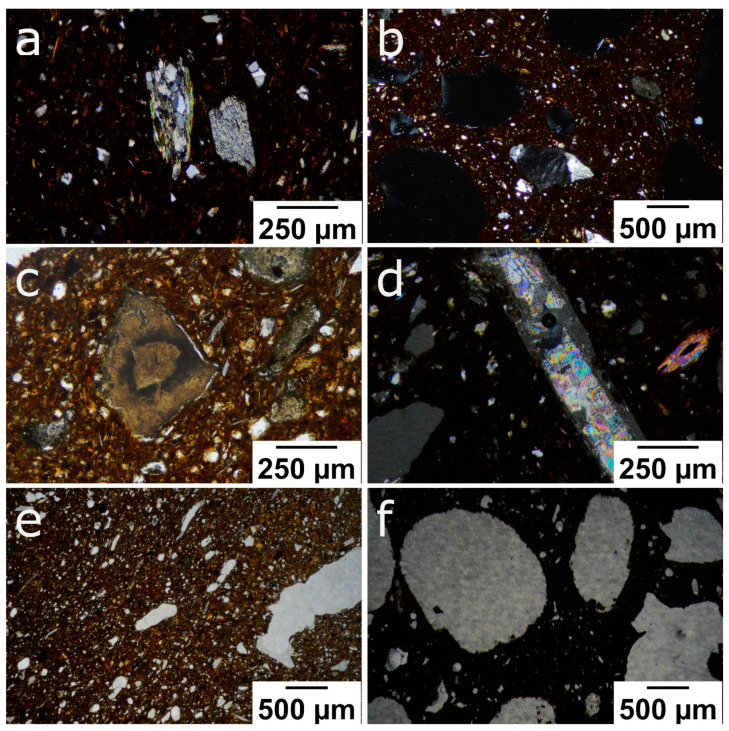
Polarised optical microscope images of the following: (**a**) gneiss fragments and small quartz grains in a brownish matrix (R, PPX); (**b**) feldspar grain and larger rounded pores (P6; PPX); (**c**) decomposed carbonate grain with angular morphology (P2; PPL); (**d**) elongated anhydrite fragment with high interference colour (P6; PPX); (**e**) general view of the porosity of control sample (R, PPL); (**f**) shape of the pores in a brick made with EPS (P6, PPL). Abbreviation: PPL = plane-polarised light; PPX = cross-polarised light.

As can be observed through the polarised optical microscope, all the samples had a brownish, low birefringent matrix. The temper of the bricks was made up of gneiss fragments (Figure 4a), quartz grains with an angular morphology and undulatory extinction, and fragments of K-feldspar (Figure 4b). Muscovite crystals with up to a second-order blue interference colour appear as isolated grains or as a component of the gneiss fragments together with quartz. Some decomposed brownish-coloured carbonate grains (Figure 4c) and anhydrite fragments (Figure 4d) can also be observed. The anhydrite formed due to the dehydration of the gypsum crystals contained in the raw material. Regarding the texture of the fired bricks, the sample without additives contains elongated pores (Figure 4e), while those made with additives had larger, more abundant pores with a more rounded shape (Figure 4f). This last characteristic was more noticeable in the bricks in which EPS was used as an additive.

**Figure 5:**
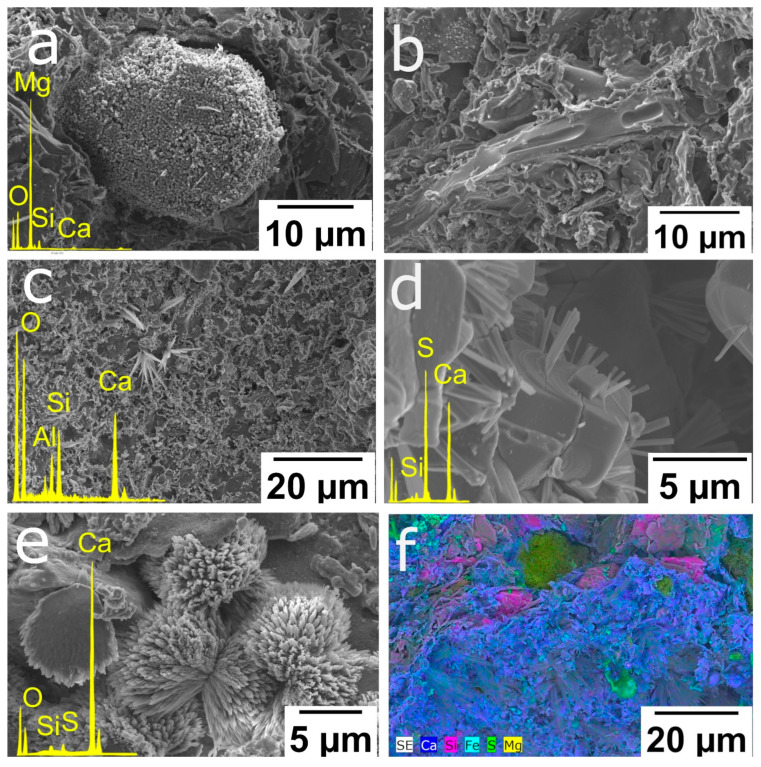
ESEM secondary electron images of fired bricks: (**a**) rounded Mg oxide from a former dolomite grain with the presence of nanocrystals on the surface (P2); (**b**) view of partially vitrified matrix in which smooth surfaces and rounded pores can be seen (H2); (**c**) micrometric gehlenite crystals covering a pore surface (P2); (**d**) development of secondary gypsum crystals on anhydrite grains (H2); (**e**) growth of secondary calcite crystals with scalenohedral habit (H6); (**f**) compositional map of a section close to a sample pore (P2).

Figure 5 shows various microtexture features of the bricks made with and without additives as viewed through ESEM. Rounded grains identified as Mg oxides could also be observed (Figure 5a). Note the presence of very small pores on the surface of the grain. Rodriguez-Navarro et al. [44] indicated that during dolomite firing, an aggregate of anhedral MgO and CaO nanocrystals appeared on the surface of the former carbonate grain, which gave it its porous appearance. In addition, the decomposition of dolomite fragments during firing leads to the incorporation of Ca ions into the gehlenite lattice, while Mg remains in the grain due to the fact that it is less mobile [45]. The brick matrix is partially vitrified, causing smooth surfaces to develop and pores to coalesce, assuming ellipsoidal morphology (Figure 5b). Some gehlenite crystals were observed covering the surface of large pores (Figure 5c). Figure 5d suggests the formation of acicular gypsum crystals after rehydration of the anhydrite. As mentioned above, anhydrite is formed during firing, but the sample’s later immersion in water to prevent “lime blowing” (see Section 2.1 making the brick samples) may have led to the partial rehydration of anhydrite grains and the development of gypsum crystals [46]. Similarly, during immersion, secondary calcite with dogtooth morphology also formed (Figure 5e). Figure 5f shows a compositional map of sample P2 where gypsum/anhydrite crystals are identified (green, upper left corner) as well as Mg oxides (yellow, centre), phyllosilicates (pink) and gehlenite crystals (purple, covering a pore).

### 3.2. Study of the Porous System

**Figure 6:**
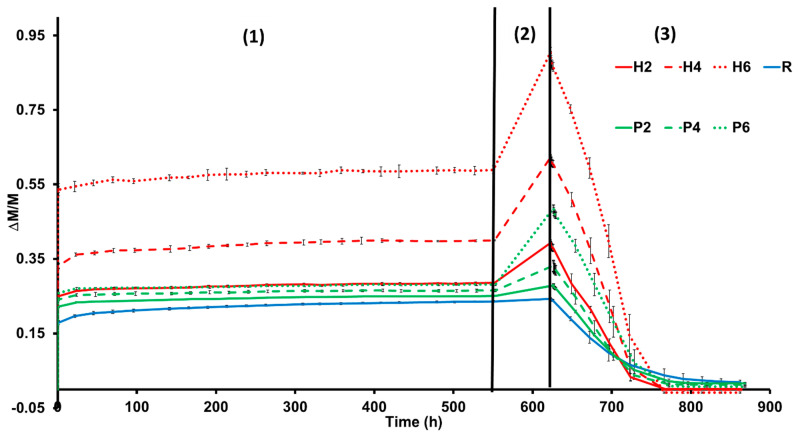
Free water absorption (1), forced water absorption (2) and drying (3) of bricks made without additives (R), with the addition of EPS (P2, P4 and P6) or olive stones (H2, H4 and H6). Weight variation (ΔM/M) against time (in hours). Each curve represents the mean of three measurements. Brick abbreviations are indicated in Table 1.

The porous system was analysed using hydric tests and MIP. The results of this analysis are summarised in Figure 6 and Table 3.

Samples with added EPS or added olive stones absorb more water under free and forced absorption (Ab and Af, respectively, Table 3 and Figure 6) compared to the bricks without additives. The bricks with additives also show lower pore interconnectivity (higher Ax, Table 3), which leads to lower water saturation (S, Table 3). In addition, the samples with additives dry faster (D_i_, Table 3) despite having lower pore interconnectivity values (Ax). This can be explained by the roughness of the faces of the samples made with additives, which increases the surface exposure to the air, favouring a quicker dry [47]. According to Hall and Hoff [47], the drying process consists of two stages. The first (constant drying rate) is not related to the porosity of the material and is dependent on external conditions. By the end of the drying test, samples H4 and H6 weighed less than at the beginning (Figure 6). This indicates that the samples containing olive stones are more prone to losing fragments during contact with water.

As expected [14,17], the addition of both additives increased the open porosity of the samples (P_o_, Table 3) and decreased their apparent density (ρ_a_, Table 3). However, the real density (ρ_r_, Table 3) of each sample is unrelated to its porosity and depends exclusively on the mineralogy.

**Figure 7:**
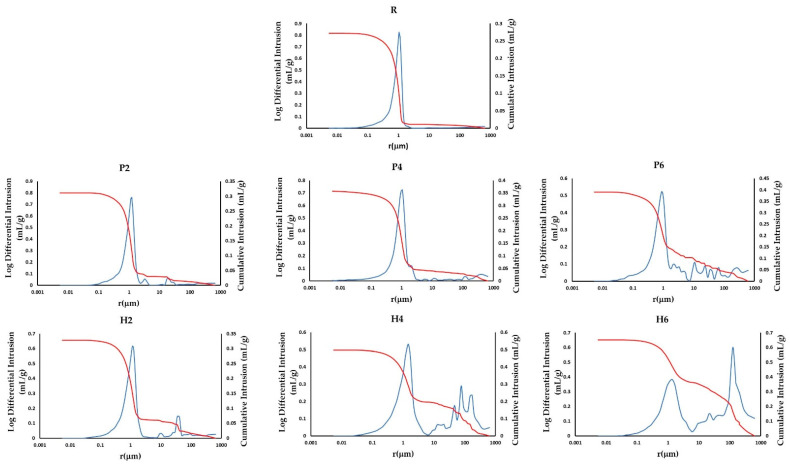
Cumulative mercury intrusion (red) and pore size distribution (blue) curves for the samples without additives (R) and for those with added EPS (P) and olive stones (H).

The MIP results confirm those obtained in the hydric tests. The addition of the two waste materials increases the porosity of the bricks (P_o(MIP),_ Table 3), especially with the addition of olive stones, and decreases their apparent density (ρ_a(MIP),_ Table 3). In addition, the pore size distribution and cumulative mercury intrusion curves (Figure 7) reveal that the sample without additives has a unimodal distribution, with a maximum peak at around 1 μm (R, Figure 7). On the other hand, the presence of additives favours the creation of new pore families to the right of the curve (i.e., pores with a higher radius). Specifically, the addition of EPS leads to the formation of new families with a radius of 3, 10 and 100 μm (P2, P4 and P6, Figure 7), while the addition of olive stones generates new families with a radius of 10, 50 and 100 μm (H2, H4 and H6, Figure 7). It is evident that the size of these new pore families increases as the percentage of additives augments, especially for the H samples.

### 3.3. Compactness, Colour and Thermal Conductivity

**Figure 8:**
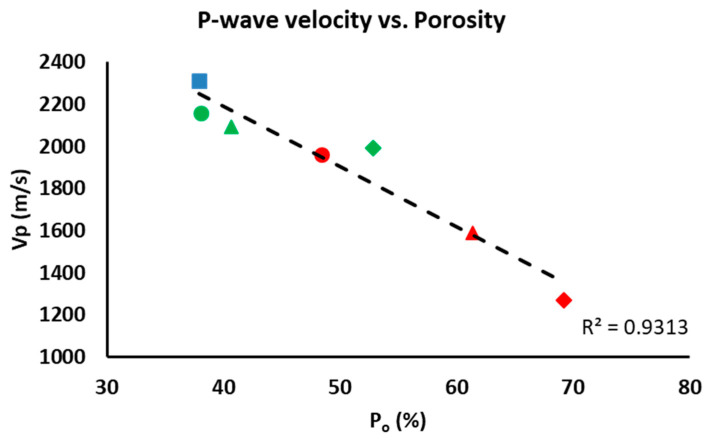
Comparison between the open porosity of the samples (P_o_) and the P-wave velocity (Vp). This figure shows control samples (blue square), samples with EPS (green) and samples with olive stones (red) at 20% (circle), 40% (triangle) and 60% (diamond).

The P-wave velocity is mainly related to the mineralogy of the samples, their orientation, pores and fissures [48]. As mentioned earlier, there are no significant mineralogical differences between the samples, which means that the ultrasound results will depend exclusively on the porosity. In fact, P-wave velocity (Vp) is lower in samples with a higher percentage of additive due to their higher porosity (see P_o_, Table 3). Consequently, there is an inverse relationship between additive content and Vp. Figure 8 compares the open porosity (P_o_) obtained from the hydric tests with the Vp from the ultrasound test, resulting in a linear trend with a high correlation coefficient (R^2^ = 0.9313). The control samples were more compact than the bricks made with additives. The compactness of the brick samples declined as the amount of additive increased. If we compare the samples made with the two different additives, the ones with olive stones had the lowest compactness values.

**Figure 9:**
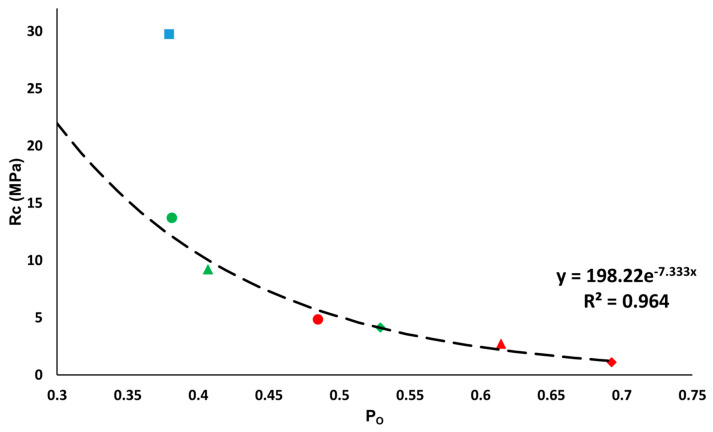
Relationship between the uniaxial compression resistance (Rc, in MPa) and the open porosity (P_o_, per unit) obtained by the hydric tests. This figure represents control samples (blue square), samples with EPS (green) and samples with olive stones (red) at 20% (circle), 40% (triangle) and 60% (diamond). The trendline is calculated for the samples with additives.

Regarding mechanical strength, the bricks without additives are the most resistant, reaching 29.75 MPa, followed by the samples with EPS and those with olive stones (Figure 9). The higher the proportion of additives, the lower the resistance of the bricks. The least resistant brick was H6 with 1.11 MPa (Figure 9). According to Spanish Building Industry Standard RL-88 [49], 10 MPa is the minimum compressive resistance value recommended for construction work with bricks. The only bricks to exceed this value were the control sample (R) and the sample with 20% EPS (P2, 13.74 MPa) (Figure 9). However, in the standard governing lightweight ceramics [50], the minimum required falls to 5 MPa. This minimum threshold was achieved by the samples with 40% of EPS (P4, 9.19 MPa), while those made with 20% of olive stones almost reached it (H2, 4.84 MPa). Figure 9 compares the results for open porosity (P_o_) obtained from the hydric tests with those from the uniaxial compression test (Rc). An exponential trendline can be drawn for the samples with additives. The associated equation is defined by the Ryshkewitch formula [51], which relates the compressive strength (σ) of the sample with its porosity (p):

(2) $$\sigma =\sigma_{0}e^{-kp}$$

where $\sigma_{0}$ is the compressive strength value of an ideal sample with no pores. The $\sigma_{0}$ value is related to the tensile fracture strength of mullite, which is between 150 and 220 MPa [52] (in our case $\sigma_{0}$ is 198.22), and k is an empirical constant between 6 and 9 (in our case 7.33).

All the samples with additives conform to the trendline defined by the previous formula. However, the control sample does not conform to this trendline because of its smaller pore size [53].

In terms of colour, the samples with no additives and those with EPS show the same lightness and chromatic parameters, despite having different porosity (Table 4). By contrast, the bricks manufactured with olive stones are lighter (higher L*, Table 4) and less red-coloured (lower a*, Table 4). They also had a lower chroma (C*) and a higher hue (h°), which also indicate their less reddish colour. The colour difference (ΔE) between the samples with additives and the control samples is always higher for H samples. This is mainly due to the smaller amounts of hematite detected in these samples by XRD and the fact that they have larger pores, which alter the visual appearance of the surface [54]. In fact, H samples have a ΔE value of over 5, which means that a standard observer could notice the difference in colour between them and the control samples [55]. By contrast, the P samples showed ΔE values between 1 and 2, which means that any colour difference between them and the control samples could only be observed by experts.

**Figure 10:**
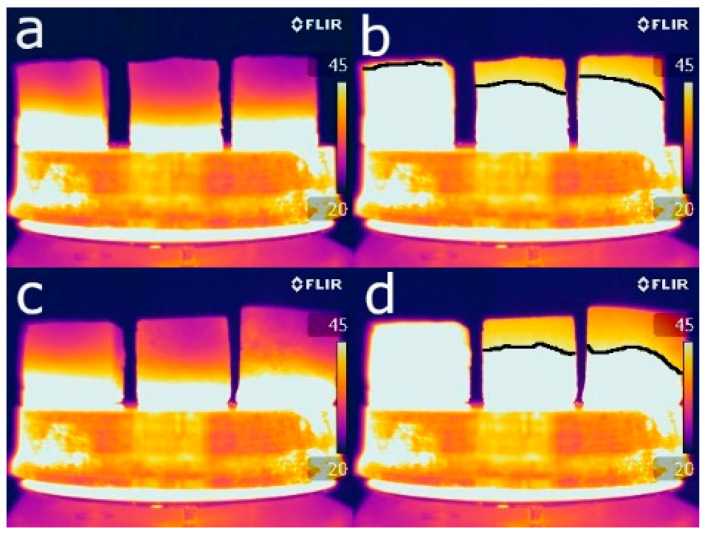
Thermographic images of the control samples (on the left in each image), the samples with EPS (centre) and the samples with olive stones (right), using 20% volume of additive (**a**,**b**) and 60% (**c**,**d**). Images were taken after 5 min (**a**,**c**) and 30 min (**b**,**d**) of heating.

Heat transfer within construction materials depends mainly on their porosity, mineralogical composition, and the fluids contained in their pores [56]. Given that all the bricks have almost the same mineralogical composition and that the measurements were taken on dried samples, porosity is the main factor affecting the infrared thermography results. Figure 10 shows the thermographic images obtained 5 min (Figure 10a,c) and half an hour (Figure 10b,d) after the beginning of the test. These show that after the first 5 min, the isotherms were at the same height in all the samples. As the test progressed, the height of the isotherm in the control sample increased more quickly than in the samples with additives. There is, therefore, an inverse relationship between additive content and thermal conductivity due to the presence of pores which slow down the transmission of heat inside the bricks, thus improving their thermal insulation [57].

### 3.4. Durability by Salt Crystallisation

**Figure 11:**
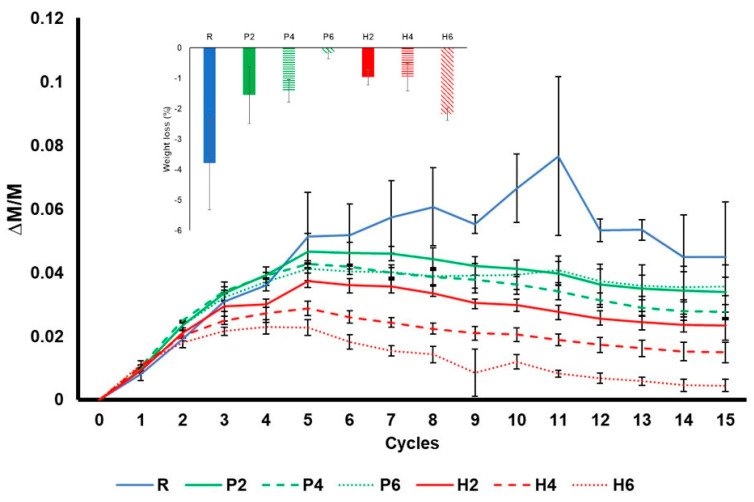
Weight variation in the bricks without additives (R), with EPS (P) and with olive stones (H) over 15 cycles of the salt crystallisation decay test. Each cycle represents 24 h, and each curve is a mean of 3 measurements. The error bars represent the standard deviation. The bar chart above shows the weight loss in the samples at the end of the test.

Figure 11 shows the durability of bricks to salt crystallisation during the 15 test cycles. At the beginning of the test, all the samples gain weight due to the crystallisation of sodium sulphate in their pore system. The samples without additives (R) underwent the greatest increase in weight and the greatest oscillation, while the samples with olive stones registered the smallest increase. This is because the R samples have better interconnectivity between the pores (see Ax, Table 3), which means that the solution can easily reach the deeper pores in these samples.

If we analyse this graph in detail, after the initial increase, the samples with additive began losing weight from cycle 5, while the weight of the R samples continued increasing until cycle 8. The weight of the R samples fluctuates considerably, with a high standard deviation (Figure 11). The fluctuations in weight observed during this ageing test can be explained as follows: (1) sodium sulphate starts occupying the pore system of bricks, causing the weight of the samples to increase; (2) the dissolution and recrystallisation of salt in confined spaces (pores and fissures) causes the development of fractures [58], the loss of fragments and a decrease in weight; (3) the appearance of new pores and fissures allows new brick material to be exposed to the saline solution, causing an increase in weight, and the process repeats itself. In addition, although the loss of fragments in the R samples was less frequent than in the rest of the samples when it occurred, larger fragments were lost. In fact, while P and H samples suffered powdering, R samples suffered crumbling.

At the end of the test, the samples were washed to remove the remaining salts and study the weight loss that took place during the test. The R bricks suffered the highest weight loss. According to Benavente et al. [59], the resistance of the samples to salt decay is directly related to their compressive strength and P-wave velocity, which means that the R samples should have been the most durable bricks in this test. The fact that they suffered the greatest weight loss may be due to their smaller pore size compared to the bricks with additives. This is because large pores allow salts to crystallise inside them but prevent them from exerting pressure on the pore walls and breaking them [60]. This is why rocks with large pores, such as travertines, are more resistant to salt crystallisation because their large empty spaces act as sinkholes, reducing the concentration of the solution in the rock pores [61]. Regarding the samples with additives, the weight loss was similar in all of them except for H6. The high weight loss registered in H6 is related to its brittleness, as observed at the end of the drying test (see Figure 6).

Finally, the addition of both wastes has some advantages in the resulting bricks, such as the increase in thermal insulation and in salt crystallisation resistance, but also bares some drawbacks, for example, the decrease in compressive resistance. Table 5 summarises the main mineralogical, physical and mechanical results obtained in this research.

## 4. Conclusions

In this paper, we studied the effects of the addition of expanded polystyrene (EPS) and olive stones to handmade bricks. To this end, we compared the mineralogical, textural and physical properties of these bricks with those of the control samples made with the same clayey material and fired at the same temperature but without additives. 

The addition of both additive products (expanded polystyrene and olive stone) increased the porosity of the fired bricks and their thermal insulation and decreased their apparent density. The addition of olive stone diminishes the reddish colour of the bricks because a smaller amount of hematite is developed in these samples. 

Regarding the resistance of the bricks to the uniaxial compression test and decay test, samples with a high percentage of additives showed better resistance to salt crystallisation and less compressive resistance. In fact, only the R and P2 samples exceeded the limits recommended for construction work with bricks (10 MPa). For its part, the minimum compressive resistance for lightweight bricks (5 MPa) was also achieved by the P4 sample and almost achieved by the H2 sample. Despite having similar open porosity values as samples with 20% EPS, the samples without additives had higher compressive strength due to their smaller pore size (as verified by the MIP results). Consequently, the compressive resistance of the bricks manufactured with additives could perhaps be improved by grinding them more finely, reducing their pore size, before mixing them with the clayey earth. However, the pore size explains the better durability of the samples with additives, as it is more difficult for the salts to exert pressure and cause damage in the confined spaces of large pores. 

The use of these additives in brick production offers several advantages, such as the light weight of the bricks and their good thermal insulation. However, the fact that they have lower compressive strength is an important drawback. This could perhaps be solved or minimised by grinding the waste products more finely so as to reduce their pore size before mixing them with the clay. Further research needs to be conducted to verify this possibility, especially in the case of olive stones.

## Figures and Tables

**Table 1:** Sample abbreviations, percentage of additive used (by volume and by mass with respect to samples with no additive), and amount of water used during the moulding process in each brick.

Temperature	Additive	% Volume	% Mass	Water Used (mL)	Sample Name
950 °C	Without additive	-	-	500	R
	Expanded polyethylene (EPS)	20	0.3	500	P2
		40	0.7	475	P4
		60	1.1	450	P6
	Olive stones	20	13.2	400	H2
		40	25.6	350	H4
		60	38.5	325	H6

**Table 2:** Chemical analysis of the major oxides (in wt.%) in the raw material from Viznar and the fired bricks. LOI stands for loss of ignition. Sample abbreviations as in Table 1.

Sample	SiO_2_	Al_2_O_3_	Fe_2_O_3_	MnO	MgO	CaO	Na_2_O	K_2_O	TiO_2_	P_2_O_5_	LOI
Viznar	48.25	15.77	5.69	0.08	2.88	10.10	0.79	2.82	0.74	0.13	12.02
R	53.30	17.66	6.30	0.09	3.23	11.68	0.88	3.12	0.82	0.15	1.32
P2	53.65	17.64	6.23	0.08	3.28	11.72	0.87	3.11	0.85	0.14	1.82
P4	53.59	17.67	6.25	0.09	3.27	11.71	0.92	3.15	0.85	0.15	1.72
P6	53.33	17.65	6.22	0.08	3.23	11.65	0.87	3.11	0.85	0.14	1.76
H2	53.44	17.69	6.21	0.09	3.33	11.70	0.89	3.19	0.83	0.15	1.63
H4	53.87	17.75	6.34	0.09	3.23	11.26	0.92	3.25	0.84	0.15	1.53
H6	53.37	17.53	6.22	0.08	3.25	11.36	0.86	3.26	0.84	0.15	1.44

**Table 3:** Hydric parameters and MIP results for bricks with no additives (R), with EPS (P2, P4 and P6) and olive stones (H2, H4 and H6). Ab: free water absorption (%); Af: forced water absorption (%); Ax: interconnection between pores (%); Di: drying index (%); S: saturation coefficient (%); P_o_: open porosity (%); ρ_a_: apparent density (g/cm^3^); ρ_r_: real density (g/cm^3^); P_o(MIP)_: open porosity obtained through MIP (%); ρ_a(MIP)_: apparent density obtained through MIP (g/cm^3^); ρ_r(MIP)_: real density obtained through MIP (g/cm^3^).

	R	P2	P4	P6	H2	H4	H6
A_b_	23.54	25.01	26.47	28.10	28.51	40.27	58.90
A_f_	24.21	27.84	33.19	48.64	39.30	62.54	90.60
A_x_	2.78	10.13	20.16	42.23	27.45	35.60	34.98
D_i_	0.89	0.87	0.86	0.83	0.84	0.80	0.77
S	84.26	84.64	76.52	55.58	68.23	58.54	61.16
P_o_	37.94	38.12	40.71	52.91	48.46	61.44	69.28
ρ_a_	1.57	1.37	1.23	1.09	1.23	0.98	0.76
ρ_r_	2.53	2.21	2.07	2.31	2.39	2.55	2.49
P_0(MIP)_	40.43	44.67	46.35	47.74	44.12	53.66	58.89
ρ_a(MIP)_	1.48	1.43	1.29	1.22	1.34	1.08	0.90
ρ_r(MIP)_	2.49	2.59	2.42	2.34	2.40	2.33	2.20

**Table 4:** Lightness (L*), chromatic values (a* and b*), chroma (C*), hue angle (h°) and the colour difference (ΔE) between the control samples (R) and those with additives (P and H).

	L*	a*	b*	C*	h°	ΔE*
R	54.51	21.60	28.82	36.02	53.14	-
P2	54.64	21.13	27.90	35.00	52.85	1.04
P4	55.23	20.53	27.95	34.68	53.69	1.55
P6	55.03	20.92	28.27	35.17	53.49	1.02
H2	58.55	18.20	28.21	33.66	57.47	5.32
H4	59.13	14.07	25.70	29.34	61.26	9.37
H6	59.95	13.77	26.08	29.50	62.11	9.92

**Table 5:** Mineralogical and physical–mechanical differences and similarities between the bricks made with added expanded polystyrene (P), added olive stones (H) and the control samples with no additives. An upward arrow indicates that the sample had a higher value for this parameter than the control sample. A downward arrow indicates that the sample had a lower value for this parameter than the control sample. The number of arrows indicates the relative variation with regard to the control sample.

	P2	P4	P6	H2	H4	H6
Mineralogical changes	NO	NO	NO	YES	YES	YES
Porosity	↑	↑↑	↑↑↑	↑	↑↑	↑↑↑
Pore size distribution	1, 3, 10 and 100 μm	1, 3, 10 and 100 μm	1, 3, 10 and 100 μm	1, 10, 50 and 100 μm	1, 10, 50 and 100 μm	1, 10, 50 and 100 μm
Tortuosity	↑	↑↑	↑↑↑	↑	↑↑	↑↑↑
Drying speed	↑	↑↑	↑↑↑	↑	↑↑	↑↑↑
Apparent density	↓	↓↓	↓↓↓	↓	↓↓	↓↓↓
P-wave velocity	↓	↓↓	↓↓↓	↓	↓↓	↓↓↓
Compressive strength	↓	↓↓	↓↓↓	↓	↓↓	↓↓↓
Colour change	NO	NO	NO	YES	YES	YES

## Data Availability

The data presented in this study are available on request from the corresponding author.

## References

[1] Al-Fakih A., Mohammed B.S., Liew M.S., Nikbakht E. (2019). Incorporation of Waste Materials in the Manufacture of Masonry Bricks: An Update Review. J. Build. Eng..

[2] Murmu A.L., Patel A. (2018). Towards Sustainable Bricks Production: An Overview. Constr. Build. Mater..

[3] Zhang L. (2013). Production of Bricks from Waste Materials—A Review. Constr. Build. Mater..

[4] Sutcu M., Alptekin H., Erdogmus E., Er Y., Gencel O. (2015). Characteristics of Fired Clay Bricks with Waste Marble Powder Addition as Building Materials. Constr. Build. Mater..

[5] Yang C., Cui C., Qin J., Cui X. (2014). Characteristics of the Fired Bricks with Low-Silicon Iron Tailings. Constr. Build. Mater..

[6] Cultrone G. (2022). The Use of Mount Etna Volcanic Ash in the Production of Bricks with Good Physical-Mechanical Performance: Converting a Problematic Waste Product into a Resource for the Construction Industry. Ceram. Int..

[7] Hasan R., Siddika A., Akanda P.A., Islam R. (2020). Effects of Waste Glass Addition on the Physical and Mechanical Properties of Brick. Innov. Infrastruct. Solut..

[8] Görhan G., Şimşek O. (2013). Porous Clay Bricks Manufactured with Rice Husks. Constr. Build. Mater..

[9] Muñoz P., Letelier V., Zamora D., Morales M.P. (2020). Feasibility of Using Paper Pulp Residues into Fired Clay Bricks. J. Clean. Prod..

[10] Anape Medio Ambiente. http://www.reciclado-eps.com/index.php.

[11] Xi G., Liang R., Tang Q., Li J. (1999). Mechanism Studies on the Catalytic Degradation of Waste Polystyrene into Styrene in the Presence of Metal Powders. J. Appl. Polym. Sci..

[12] EUMEPS Submitted Voluntary Pledge—EUMEPS. https://eumeps.org/news/eumeps-submitted-voluntary-pledge.

[13] Ali Y.A.Y., Fahmy E.H.A., AbouZeid M.N., Shaheen Y.B.I., Mooty M.N.A. (2020). Use of Expanded Polystyrene in Developing Solid Brick Masonry Units. Constr. Build. Mater..

[14] Veyseh S., Yousefi A.A. (2003). The Use Of Polystyrene In Lightweight Brick Production. Iran. Polym. J..

[15] Ramezani A., Nemat S., Emami S.M. (2018). Effects of the Size of Expanded Polystyrene as a Pore-Former on the Properties of Insulating Firebricks. Ceram. Int..

[16] Bernal E.M., Vlasova M., Márquez A.A.P., Kakazey M., Tapia R.G. (2020). Synthesis and Properties of Porous Bricks Obtained with the Use of Spherical Expanded Polystyrene Particles of Packaging Material. Sci. Sinter..

[17] La Rubia-García M.D., Yebra-Rodríguez Á., Eliche-Quesada D., Corpas-Iglesias F.A., López-Galindo A. (2012). Assessment of Olive Mill Solid Residue (Pomace) as an Additive in Lightweight Brick Production. Constr. Build. Mater..

[18] Pérez-Villarejo L., Eliche-Quesada D., Martín-Pascual J., Martín-Morales M., Zamorano M. (2020). Comparative Study of the Use of Different Biomass from Olive Grove in the Manufacture of Sustainable Ceramic Lightweight Bricks. Constr. Build. Mater..

[19] Eliche-Quesada D., Iglesias-Godino F.J., Pérez-Villarejo L., Corpas-Iglesias F.A. (2014). Replacement of the Mixing Fresh Water by Wastewater Olive Oil Extraction in the Extrusion of Ceramic Bricks. Constr. Build. Mater..

[20] Aouba L., Bories C., Coutand M., Perrin B., Lemercier H. (2016). Properties of Fired Clay Bricks with Incorporated Biomasses: Cases of Olive Stone Flour and Wheat Straw Residues. Constr. Build. Mater..

[21] Arezki S., Chelouah N., Tahakourt A. (2016). The Effect of the Addition of Ground Olive Stones on the Physical and Mechanical Properties of Clay Bricks. Mater. Constr..

[22] El Boukili G., Ouakarrouch M., Lechheb M., Kifani-Sahban F., Khaldoune A. (2021). Recycling of Olive Pomace Bottom Ash (by-Product of the Clay Brick Industry) for Manufacturing Sustainable Fired Clay Bricks. Silicon.

[23] Eliche-Quesada D., Leite-Costa J. (2016). Use of Bottom Ash from Olive Pomace Combustion in the Production of Eco-Friendly Fired Clay Bricks. Waste Manag..

[24] Evaluación de la Producción Y Usos de Los Subproductos de las Agroindustrias del Olivar en Andalucía. Observatorio de Precios Y Mercados. Consejería de Agricultura, Pesca Y Desarrollo Rural. Junta de Andalucía. https://www.juntadeandalucia.es/agriculturaypesca/observatorio/servlet/FrontController?action=RecordContent&table=11031&element=1585171&.

[25] Braga J.C., Martín J.M., Quesada C. (2003). Patterns and Average Rates of Late Neogene–Recent Uplift of the Betic Cordillera, SE Spain. Geomorphology.

[26] Braga J.C., Martin J.M., Alcala B. (1990). Coral Reefs in Coarse-Terrigenous Sedimentary Environments (Upper Tortonian, Granada Basin, Southern Spain). Sediment. Geol..

[27] Dabrio C.J., Fernández Martínez J., Dabrio C.J., Fernández Martínez J. (1986). Depósitos de ríos trenzados conglomeráticos Plio-Pleistocénicos de la Depresión de Granada. Cuad. Geol. Ibérica.

[28] Knapek M., Húlan T., Minárik P., Dobroň P., Štubňa I., Stráská J., Chmelík F. (2016). Study of Microcracking in Illite-Based Ceramics during Firing. J. Eur. Ceram. Soc..

[29] Cultrone G., Rodriguez-Navarro C., Sebastian E., Cazalla O., De La Torre M.J. (2001). Carbonate and Silicate Phase Reactions during Ceramic Firing. Eur. J. Mineral..

[30] (2008). Métodos de Ensayo Para Piedra Natural. Determinación de La Absorción de Agua a Presión Atmosférica.

[31] (1988). Misura Dell’indice Di Asciugamento (Drying Index).

[32] RILEM (1980). Recommended Test to Measure the Deterioration of Stone and to Assess the Differences of Treatment Methods. Mater. Struct..

[33] (1999). Métodos de Ensayo de Piezas Para Fábrica de Albañilería. Parte 4: Determinación de La Densidad Real y Aparente y de La Porosidad Abierta y Total de Piezas de Piedra Natural Para Fábrica de Albañilería.

[34] Cultrone G., Sebastián E., Elert K., de la Torre M.J., Cazalla O., Rodriguez–Navarro C. (2004). Influence of Mineralogy and Firing Temperature on the Porosity of Bricks. J. Eur. Ceram. Soc..

[35] (2008). Standard Test Method for Laboratory Determination of Pulse Velocities and Ultrasonic Elastic Constant of Rock.

[36] (2007). Métodos de Ensayo Para La Piedra Natural. Determinación de La Resistencia a La Compresión Uniaxial.

[37] (2014). Pigmentos Para La Coloración de Materiales de Construcción Fabricados a Partir de Cemento y/o Cal. Especificaciones y Métodos de Ensayo.

[38] (1999). Métodos de Ensayo Para Piedra Natural. Determinación de La Resistencia a La Cristalización de Las Sales.

[39] Saenz N., Sebastián E., Cultrone G. (2019). Analysis of Tempered Bricks: From Raw Material and Additives to Fired Bricks for Use in Construction and Heritage Conservation. Eur. J. Mineral..

[40] Warr L.N. (2021). IMA–CNMNC Approved Mineral Symbols. Mineral. Mag..

[41] Nodari L., Marcuz E., Maritan L., Mazzoli C., Russo U. (2007). Hematite Nucleation and Growth in the Firing of Carbonate-Rich Clay for Pottery Production. J. Eur. Ceram. Soc..

[42] Cultrone G., Carrillo Rosua F.J. (2020). Growth of Metastable Phases during Brick Firing: Mineralogical and Microtextural Changes Induced by the Composition of the Raw Material and the Presence of Additives. Appl. Clay Sci..

[43] Maritan L., Nodari L., Mazzoli C., Milano A., Russo U. (2006). Influence of Firing Conditions on Ceramic Products: Experimental Study on Clay Rich in Organic Matter. Appl. Clay Sci..

[44] Rodriguez-Navarro C., Kudlacz K., Ruiz-Agudo E. (2012). The Mechanism of Thermal Decomposition of Dolomite: New Insights from 2D-XRD and TEM Analyses. Am. Mineral..

[45] Cultrone G. (2013). Estudio Mineralógico-Petrográfico Y Físico-Mecánico de Ladrillos Macizos Para su Aplicación en Intervenciones del Patrimonio Histórico. Ph.D. Thesis.

[46] Haldar S.K., Haldar S.K. (2020). Chapter 6—Sedimentary Rocks. Introduction to Mineralogy and Petrology.

[47] Hall C., Hoff W.D. (2002). Evaporation and Drying. Water Transport in Brick, Stone and Concrete.

[48] Benavente D. (2002). Modelización Y Estimación de la Durabilidad de Materiales Pétreos Porosos Frente a la Cristalización de Sales. Ph.D. Thesis.

[49] (1988). Pliego General de Condiciones Para La Recepción de Los Ladrillos Cerámicos En Las Obras de Construcción.

[50] (2000). Bloques Cerámicos de Arcilla Aligerada. Designación y Especificaciones.

[51] Chen X., Wu S., Zhou J. (2013). Influence of Porosity on Compressive and Tensile Strength of Cement Mortar. Constr. Build. Mater..

[52] Hattiangadi A., Bandyopadhyay A. (2000). Strength Degradation of Nonrandom Porous Ceramic Structures under Uniaxial Compressive Loading. J. Am. Ceram. Soc..

[53] Le Huec J.C., Schaeverbeke T., Clement D., Faber J., Le Rebeller A. (1995). Influence of Porosity on the Mechanical Resistance of Hydroxyapatite Ceramics under Compressive Stress. Biomaterials.

[54] Simonot L., Elias M. (2003). Color Change Due to Surface State Modification. Color Res. Appl..

[55] Mokrzycki W.S., Talot M. (2021). Colour Difference ΔE. A Survey. Mach. Graph. Vis..

[56] Bhattacharjee B., Krishnamoorthy S. (2004). Permeable Porosity and Thermal Conductivity of Construction Materials. J. Mater. Civ. Eng..

[57] Cultrone G., Aurrekoetxea I., Casado C., Arizzi A. (2020). Sawdust Recycling in the Production of Lightweight Bricks: How the Amount of Additive and the Firing Temperature Influence the Physical Properties of the Bricks. Constr. Build. Mater..

[58] Angeli M., Bigas J.-P., Benavente D., Menéndez B., Hébert R., David C. (2007). Salt Crystallization in Pores: Quantification and Estimation of Damage. Environ. Geol..

[59] Benavente D., Linares-Fernández L., Cultrone G., Sebastián E. (2006). Influence of Microstructure on The Resistance to Salt Crystallisation Damage in Brick. Mater. Struct..

[60] Scherer G.W. (1999). Crystallization in Pores. Cem. Concr. Res..

[61] Benavente D. (2011). Why Pore Size Is Important in the Deterioration of Porous Stones Used in the Built Heritage. MACLA.

